# Periacetabular osteotomy: A novel application of modified Stoppa approach

**DOI:** 10.1051/sicotj/2022035

**Published:** 2022-08-15

**Authors:** Mostafa M. Baraka, Haitham E. Sallam, Mahmoud M. Abdelwahab

**Affiliations:** 1 Division of Paediatric Orthopaedics and Limb Reconstruction, Department of Orthopaedic Surgery, Faculty of Medicine, Ain-Shams University Abbasia 11517 Cairo Egypt; 2 Division of Hip Reconstruction, Department of Orthopaedic Surgery, Faculty of Medicine, Ain-Shams University Abbasia 11517 Cairo Egypt; 3 Division of Arthroscopy and Sports Medicine, Department of Orthopaedic Surgery, Faculty of Medicine, Ain-Shams University Abbasia 11517 Cairo Egypt

**Keywords:** Hip dysplasia, Ganz osteotomy, Periacetabular osteotomy, Anterior intrapelvic approach, Modified Stoppa approach

## Abstract

*Background*: The Bernese periacetabular osteotomy (PAO) is a well-established procedure for symptomatic hip dysplasia in adolescents and young adults. However, it remains a technically demanding procedure, and several major complications have been described, many of which are related to the approach and surgical exposure. The current study evaluates the efficacy and safety of PAO performed through a modified Stoppa approach. *Methods*: A prospective series of nine consecutive patients with hip dysplasia were treated PAO through the modified Stoppa approach. The mean age was 22.4 years (15–30 years) and the mean follow-up was 3.2 years (2–5 years). Harris hip score (HHS) was used as a functional score, and the radiographic indices included the lateral center-edge angle (LCEA) and Tönnis roof angle. *Results*: The approach allowed the osteotomy lines to be performed under direct visualization from the intra-pelvic surface of the acetabulum, aided by fluoroscopy. A lateral window was added to perform the final iliac cut and for subsequent mobilization and fixation of the acetabular fragment. The mean HHS improved significantly from 70.8 ± 4.9 points to 90.1 ± 3.3 points (*p* < 0.001). The mean LCEA improved from 8.2° ± 4.9 (range: 0–14) to 32.7° ± 5.3 (range: 26–40), with a mean improvement of 24.5°. The mean Tönnis angle improved from 28.4° ± 4.4 (range: 22–35) to 3.8° ± 3.3 (range: 0–10). Two patients had irritation from prominent screw heads that necessitated removal 1 year after the index procedure. One patient had radiographic progression of osteoarthritis. No cases of infection, non-union, heterotopic ossification, or nerve palsy were identified till the latest follow-up. *Conclusion*: Ganz PAO can be safely conducted through the modified Stoppa approach, providing direct exposure to the osteotomized surfaces, and protecting susceptible neuro-vascular structures.

*Level of evidence*: IV.

## Introduction

Acetabular dysplasia is a condition of hip instability arising from volumetric acetabular deficiency and/or maloriented acetabulum [1]. It is a recognized cause of hip pain and dysfunction in adolescents and young adults. The reported incidence is approximately 4% of the population [2]. Dysplasia and associated deformities have been recognized to be precursors for developing early hip osteoarthritis (OA) and the need for subsequent hip arthroplasty in young adults. Early recognition and treatment may delay or prevent hip joint degeneration, increase the quality of life and reduce the need for early arthroplasty [3].

The treatment of hip dysplasia remains a challenge. Since the original description of Bernese peri-acetabular osteotomy (PAO) [4], the ability to preserve the hip and its function for a substantial period is now well-established [5]. The PAO preserved the posterior column of the innominate bone, allowing for improved pelvic stability and early patient mobilization and functional recovery. The osteotomy cuts, performed very close to the joint or acetabular center of rotation, allow for significantly greater manipulation of the acetabular fragment, facilitating the complex multiplanar correction needed in patients with various degrees of dysplasia. The PAO also reliably resulted in medialization of the hip center of rotation, thus improving the biomechanics of the dysplastic hip compared to previously described techniques [4, 5].

However, PAO remains a technically demanding procedure and several major complications have been described. The occurrence of complications is greatly influenced by the surgeon’s experience and the choice of surgical approach. The most commonly utilized approach is the anterior Smith-Petersen approach, characterized by relatively extensive incisions, muscle dissection and may require muscle detachment and anterior superior iliac spine (ASIS) osteotomy [4, 6].

Several complications have been reported that are attributable to the surgical exposure, including intra-articular penetration of the hip joint, sciatic and femoral nerve palsy, acetabular osteonecrosis due to damage of blood supply from the superior gluteal artery, injury to the obturator artery, heterotopic ossification, and posterior column discontinuity, all of which arise from difficult visualization of the osteotomized bone surfaces and inability to visualize and protect susceptible anatomical neurovascular structures [7–9].

Since the original description of Bernese PAO, several modifications of the surgical approach have been utilized to reduce the operative time and morbidity of the procedure [6, 10, 11]. The anterior intrapelvic approach (modified Stoppa) has gained popularity in acetabular fractures [12, 13], allowing direct reduction and fixation of the quadrilateral surface. However, its utilization for PAO is infrequently described in previous literature. The approach can provide direct exposure to the quadrilateral surface with less muscle dissection while allowing protection of the susceptible neurovascular structures.

Two separate cadaveric studies were performed by Elmadağ et al. [14] and Akgul et al. [15] and concluded that Ganz PAO could be safely conducted utilizing the modified Stoppa approach. The approach demonstrated efficacy in the minimal dissection of muscles, direct visualization of the quadrilateral surface, maintaining the integrity of posterior column, avoiding intra-articular penetration, and protection of neurovascular structures with less image intensifier exposure.

The current study evaluated the efficacy and safety of PAO performed through a modified Stoppa approach in patients with symptomatic hip dysplasia. We hypothesized that the modified Stoppa approach could be utilized for performing PAO, allowing a minimally-invasive direct exposure of quadrilateral surface, performing the osteotomy cuts under direct vision, with less operative time, less image intensifier exposure, and less blood loss.

## Patients and methods

This prospective case series included nine consecutive patients (nine hips), conducted from September 2016 to January 2018, in a single institution. The study included two males and seven females. The mean age was 22.4 years (15–30 years). The right side was affected in four patients, and the left side in five patients. The preoperative diagnosis included developmental hip dysplasia (DDH) in seven patients. The remaining two patients had hip dysplasia associated with spastic hemiplegic cerebral palsy and both were classified as level I according to the gross motor function classification system [16].

Two patients had previous surgeries, including open reduction and femoral osteotomy in one patient with DDH and hamstring lengthening in one patient with cerebral palsy. The mean follow-up was 3.2 years (2–5 years). No patients were lost in the follow-up. Surgery was indicated for adolescents and young adults with symptomatic hip dysplasia and center-edge angle less than 15°. Hips with marked incongruency or advanced hip OA (Grade 2 or more according to Tönnis [17]) were excluded. Institutional Review Board approval was obtained prior to conducting the study.

### Clinical assessment

The preoperative assessment included history, examination and Harris hip score (HHS) [18] was used as a functional tool. The spectrum of symptoms comprised anterior (groin) pain, lateral hip pain from abductor fatigue, hip stiffness (limited range of motion), limp, and interference with activities of daily living. The examination included gait assessment, hip range of motion, and assessment of leg-length discrepancy. Anterior and posterior hip impingement tests [19] were performed to detect underlying hip impingement and related labral pathology.

### Radiographic assessment

Standard hip anteroposterior (AP) and frog-lateral radiographs were obtained and assessed for: shape and congruency of the femoral head [20], femoral neck-shaft angle [21], the lateral center-edge angle (LCEA) [20], and Tönnis roof angle [20]. Computed tomography (CT) scan was conducted to determine the three-dimensional acetabular shape and degree of version [22]. Magnetic resonance arthrography was obtained to determine the presence of labral tears and/or chondral lesions [23].

### Surgical technique

All cases were performed under general anesthesia with a preoperative dose of antibiotics. The surgical duration (defined from the time of skin incision to the end of wound closure), amount of blood loss, need for transfusion, intraoperative X-ray dosage, and length of hospital stay were assessed.

**Figure 1:**
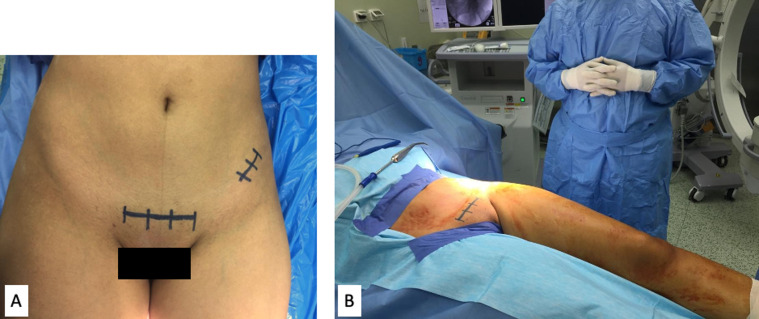
(A) Planning skin Incisions for PAO through modified Stoppa approach. The main approach through Pfannensteil incision, 10 cm long, 2 cm above the upper border of symphysis. A second lateral window, 5 cm long incision centered over ASIS to perform the iliac cut and allow for manipulation and fixation of the acetabular fragment. (B) Positioning and draping for the approach. The patient lies supine on a radiolucent table, ipsilateral leg is draped free. The fluoroscopy machine and assistant on the operated side, and the main surgeon stands on the contralateral side.

The patient was placed supine on a radiolucent table, and the ipsilateral limb was draped free to allow for necessary manipulations. A urinary catheter was placed to evacuate the bladder and facilitate intrapelvic exposure. A sterile roll was placed under the ipsilateral knee after draping to relax hip flexors and femoral neurovascular bundle and facilitate soft-tissue retraction. The fluoroscopy machine was placed on the same side of the operated pelvis, the main surgeon on the contralateral side, and the assistant on the operated side (Figure 1).

**Figure 2:**
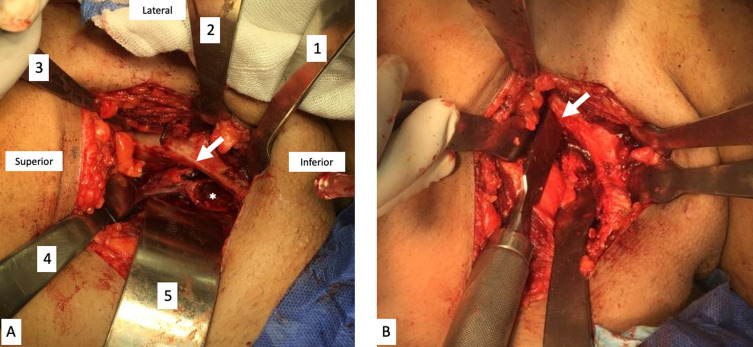
(A) Exposure of the quadrilateral surface of the left hemi-pelvis. Placement of retractors (1) behind the iliopectineal eminence, (2) middle of ilium, (3) in front of sacroiliac joint, (4) greater sciatic notch, (5) retracting peritoneum and bladder. Pelvic brim (arrow), obturator internus muscle (asterisk). (B) Performing the posterior vertical cut (arrow).

The modified Stoppa approach was performed as originally described by Cole and Bolhofner [24]. A transverse incision 10 cm in length was made 2 cm above the symphysis pubis. The rectus sheath was carefully incised in the midline along the linea alba, separating both rectus abdominis muscles. The inferior attachment of the rectus muscle on the operated side was elevated from the pubis. After the incision of the fascia transversalis, the abdominal muscles were bluntly dissected, and the peritoneum was retracted medially with a blunt retractor. The corona mortis was identified and carefully ligated. A Hohman retractor was placed anterior to the iliopectineal eminence exposing the superior pubic ramus. Second and third Hohman retractors were placed on the ilium and in front of the sacroiliac joint, respectively (Figure 2A). The iliopectineal fascia was incised at the pelvic brim, and the periosteum was elevated till a blunt Hohman retractor was inserted into the greater sciatic notch to expose the quadrilateral surface and supraacetabular region. At this stage, the obturator nerve was visualized and carefully protected. The obturator internus muscle was elevated and a Hohman retractor was placed in the lesser sciatic notch to expose the ischial ramus.

**Figure 3:**
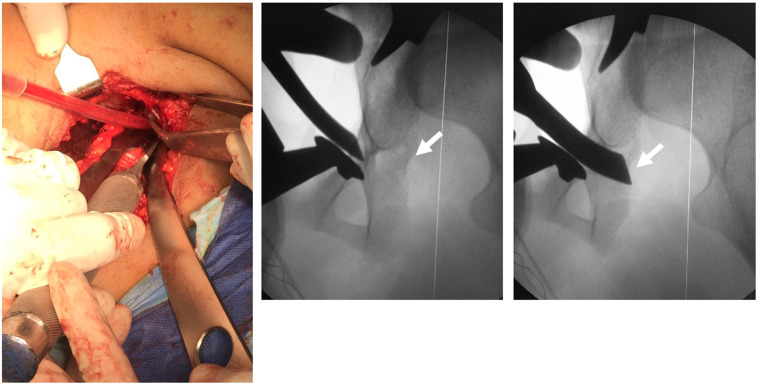
Performing the sub-cotyloid cut (arrow) under direct vision and fluoroscopic guidance.

The osteotomy was conducted as described by Ganz and Leunig [9]. The first osteotomy done is the superior pubic ramus osteotomy just medial to the iliopectineal eminence. The second osteotomy was performed along the posterior column of the acetabulum under direct visual control and fluoroscopic iliac view. This osteotomy was from the pectineal line to the inferior ischium leaving approximately 1 cm of bone posterior to the osteotomy line to preserve the posterior column (Figure 2B). Special attention was made to direct the osteotome 45° posteriorly away from the acetabulum to avoid intra-articular penetration. The third osteotomy line was the ischial osteotomy, done in the sub-cotyloid region extending to the ischial spine under direct vision and fluoroscopic guidance (Figure 3).

A separate lateral window was then performed to perform the fourth (iliac) cut and to permit mobilization and fixation of the acetabular fragment. Dissection and protection of the lateral femoral cutaneous nerve were performed. The iliac osteotomy starts 2 cm distal to the anterior superior iliac spine (ASIS) using the oscillating saw in the direction of the greater sciatic notch, but stops 1 cm before the posterior cortex to meet the posterior osteotomy line.

**Figure 4:**
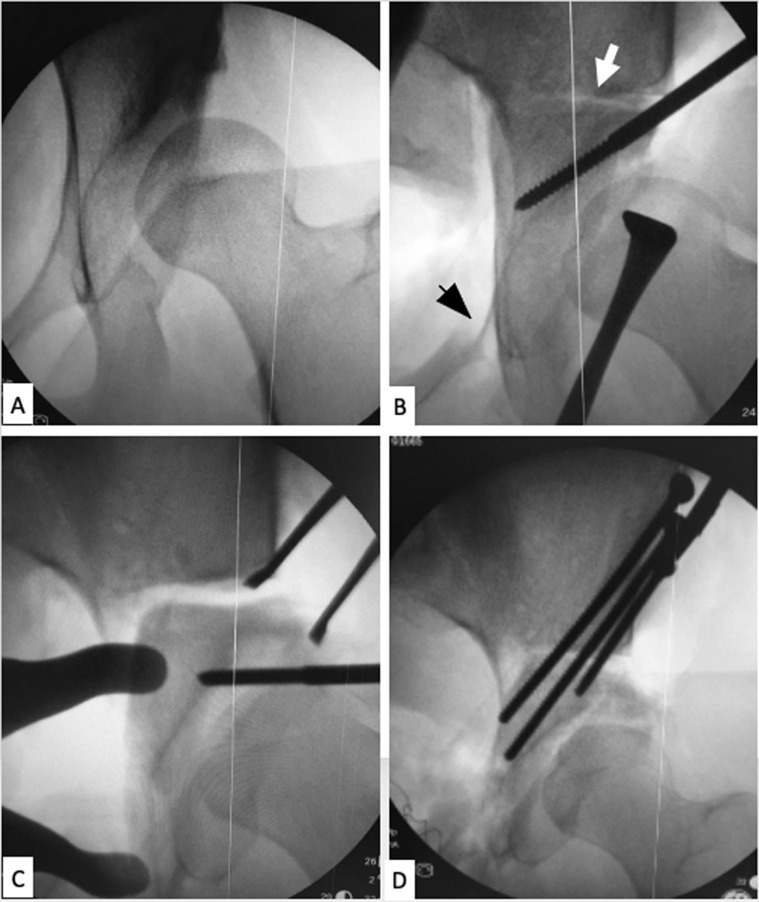
Manipulation of the acetabular fragment, re-orientation and fixation. (A) Preoperative X-ray image. (B) Upon completion of the osteotomy lines. Iliac cut (white arrow) and pubic cut (black arrow) can be seen. A 4 mm Schanz pin can be inserted into the supraacetabular area to help manipulation. (C) Fine tunning the correction with the help of a laminar spreader. (D) Final correction and fixation. Coverage (CEA) has improved with horizontal sourcil.

After completing the osteotomy lines, free mobilization of the acetabular fragment was ensured with the help of a 4 mm Schanz pin. The acetabular fragment was medialized and rotated anterolaterally using a laminar spreader. Correction aimed at a horizontal sourcil, a CEA of approximately 25–30°, with the posterior and anterior walls meeting at the lateral edge of the acetabulum (no crossover sign). The osteotomy was then fixed with 3.5-mm screws inserted through the lateral window from the iliac crest to the supraacetabular area (Figure 4).

### Postoperative protocol

All patients continued prophylactic antibiotics for 2 days, analgesics for pain control, and anticoagulant prophylaxis. They were instructed partial weight-bearing during the first 6 weeks. Radiographs were repeated monthly till osteotomy union, after which patients progressed to full weight-bearing.

All patients were re-evaluated at 6 months intervals, including clinical and functional assessment (HHS). Plain radiographs at the final follow-up included assessment union of osteotomy, radiographic indices, and observation of any radiographic OA progression (increase in Tönnis grade). Analysis of results utilized Statistical Package for Social Science (SPSS 20, IBM, Armonk, NY, USA). Analytical statistics, the McNemar test and paired *t*-test were used to assess the statistical significance.

## Results

### Radiographic parameters and clinical score

**Figure 5:**
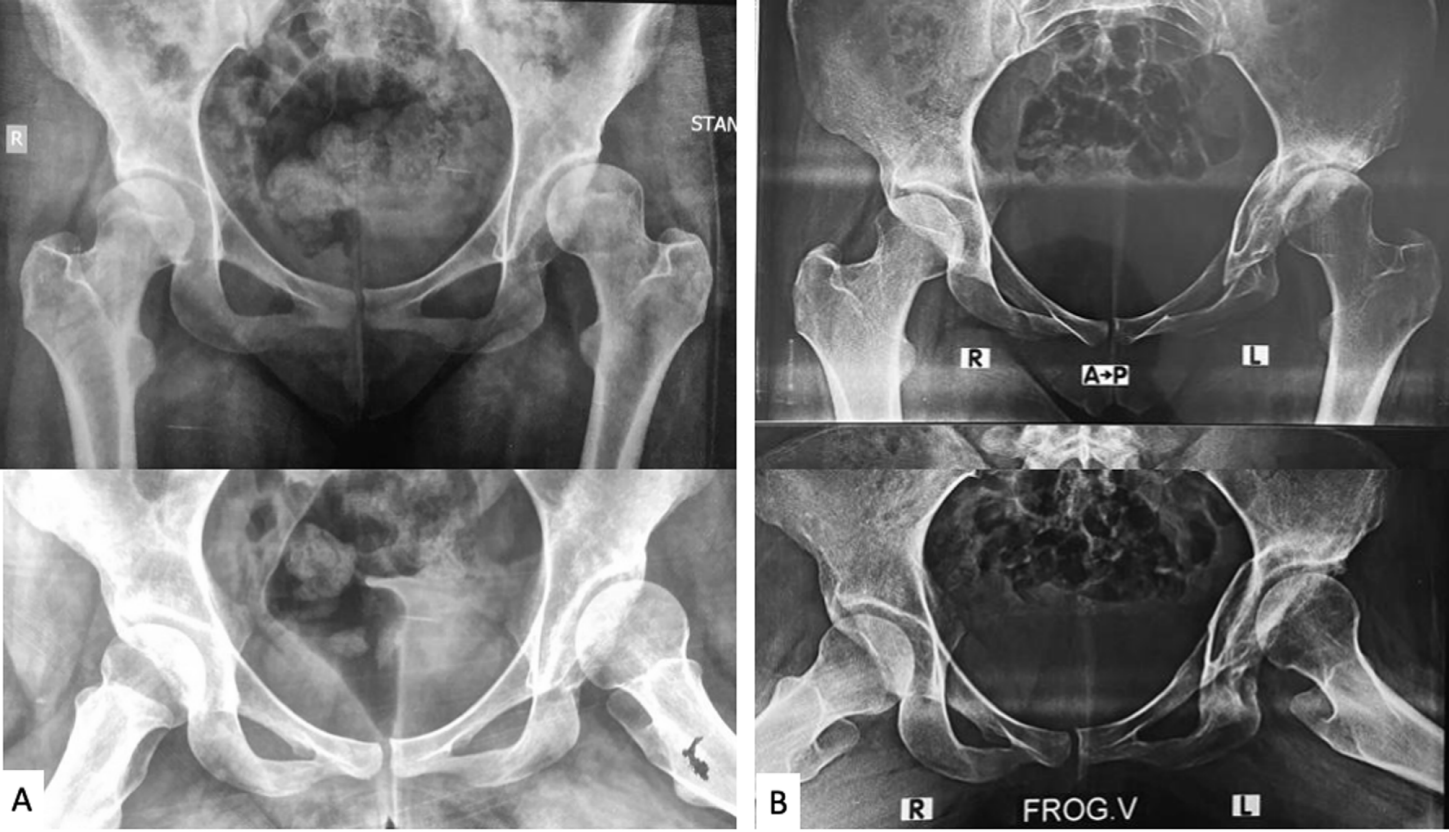
(A) Preoperative anteroposterior (AP) and frog-lateral radiographs of a 16-year-old female with left acetabular dysplasia. (B) 5-year postoperative radiograph demonstrating union of all osteotomies, maintained lateral acetabular coverage with a horizontal acetabular sourcil.

The mean HHS improved from 70.8 points to 90.1 points (*p* < 0.001). The mean LCEA improved from 8.2° (range: 0–14) to 32.7° (range: 26–40), with a mean improvement of 24.5°. The mean Tönnis angle improved from 28.4° (range: 22–35) to 3.8° (range: 0–10) (Figure 5, Tables 1 and 2). The mean estimated blood loss was 253.3 ± 79.0 mL (range: 140–360 mL). Estimated blood loss was determined by visual assessment of surgical sponges, suction canisters, and operating room environment and confirmed with the anesthesia team. The mean operative duration was 116.6 ± 37 min (75–190 min), and the mean intraoperative X-ray dose was 59.4 ± 7.2 s (50–70 s). The mean length of hospital stay was 3.5 ± 0.8 days (2–5 days).

**Table 1:** Patients’ demographic, clinical, and radiographic characteristics.

	Age	Gender	Side	Follow-up (years)	HHS – Pre (points)	HHS – Post (points)	LCEA – Pre (°)	LCEA – Post (°)	Tönnis angle – Pre (°)	Tönnis angle – Post (°)	OA degree – Pre (Tönnis grading)	OA degree – Post (Tönnis grading)	Operative time (min)	Estimated Blood Loss (ml)	Image intensifier dose (seconds)	length of hospital stay (days)
1	23	F	Rt	2	78	90	5	26	23	10	1	1	140	280	70	3
2	25	F	Rt	3	60	92	10	30	22	5	1	1	190	360	65	4
3	16	F	Lt	4	72	96	0	38	33	3	0	1	75	250	50	3
4	19	M	Lt	3	70	87	13	38	30	0	1	1	90	350	55	5
5	21	F	Lt	2	75	90	2	35	35	2	0	0	150	140	65	2
6	15	F	Lt	2	70	88	10	40	32	0	1	1	90	200	50	4
7	28	F	Rt	4	71	93	12	33	28	3	1	1	95	150	55	4
8	25	F	Lt	4	69	85	8	29	26	8	0	0	100	250	60	4
9	30	M	Rt	5	73	90	14	26	27	4	1	1	120	300	65	3

HHS: Harris hip score; LCEA: lateral center-edge angle; OA: osteoarthritis.

**Table 2:** Summary of HHS and radiographic parameters.

	Preoperative	Postoperative	Paired *t-*test	*P*-value	Significance
HHS					
Mean ± *SD*	70.89 ± 4.96	90.11 ± 3.3	−9.65	<0.001	S
Range	60–78	85–96			
LCEA (°)					
Mean ± *SD*	8.22 ± 4.92	32.78 ± 5.31	−9.33	<0.001	S
Range	0–14	26–40			
Tönnis angle (°)					
Mean ± *SD*	28.44 ± 4.45	3.89 ± 3.37	10.14	<0.001	S
Range	22–35	0–10			

HHS: Harris hip score; LCEA: lateral center-edge angle; S: significant.

### Complications

Two patients had irritation from prominent screw heads that necessitated removal 1 year after the index procedure. One patient had radiographic progression of OA in the form of narrowing of joint space width, despite an improved clinical outcome and HHS. No cases of infection, non-union, heterotopic ossification, or nerve palsy were identified till the latest follow-up.

## Discussion

Ganz PAO is a well-established procedure for the preservation of hip dysplasia [4, 5]. Several surgical approaches have been described. However, the anterior (Smith-Peterson) approach with its various modifications is the most commonly adopted [6, 10, 11]. These approaches entail extensive soft tissue dissection, provide indirect access to the osteotomized surfaces around the hip joint and capsule, and may be associated with a considerable risk of intra-articular penetration and neuro-vascular injuries. This increases the morbidity of the procedure and may be associated with significant blood loss, increased intra-operative X-ray exposure, and extended patient hospital stay.

Older reports have focused on direct exposure of the osteotomy surfaces allowing for precise periacetabular cuts. Ninomiya and Tagawa conducted a rotational PAO through combined anterior and posterior approaches without greater trochanteric osteotomy, allowing exposure to the “outer” surface of the pelvis. However, this involved extensive soft-tissue dissection of the gluteal muscles, and a Trendelenburg gait was evident after surgery and persisted in several patients [25]. Further modifications included the modified Ollier’s approach [26], and the transtrochanteric approach [27]. Broad dissection with a long skin incision and detachment of the gluteus medius muscle can result in weakness in the abduction strength of the hip.

The modified Stoppa approach has gained popularity in the pelvis and acetabular trauma surgery, providing direct access to the pelvic brim from symphysis pubis to sacroiliac joint with good exposure to quadrilateral plate [12, 13]. However, there are only a few reports utilizing this approach in PAO.

The current study shows that PAO can be performed through a modified Stoppa approach with reasonable safety and efficacy. The approach has demonstrated several advantages compared to the classic Bernese anterior approach. Providing direct exposure of the quadrilateral surface of the acetabulum allows the critical osteotomy lines, namely the posterior column and the sub-cotyloid cuts to be safely performed under direct vision rather than “blind” osteotomy techniques relying only on fluoroscopy.

Further benefits of the modified Stoppa approach are decreasing operative time, dose of X-ray exposure, and the risk of intra-articular penetration into the acetabulum. The approach provides a neat exposure with minimal muscle dissection, reducing blood loss, and morbidity of the procedure. Susceptible neuro-vascular structures; the obturator nerve and artery, corona mortis, and iliolumbar artery can be directly visualized and protected. In addition, the modified Stoppa approach may provide an opportunity to perform bilateral same-setting PAOs in cases of bilateral dysplasia. Compared to the classic anterior approach, the incisions utilized in the modified Stoppa approach are smaller, and aligned with normal creases along the bikini line. The Smith-Peterson approach may produce a less concealable vertical scar across the groin.

The initial series of the procedure by Ganz et al. [4] reported an incidence of intra-articular penetration into the acetabulum of 2.7%. In order to avoid this complication, a cadaveric study was conducted by Shiramizu et al. [28] to determine the margin of the hip from the quadrilateral surface. They recommended that the chisel inserting point should not exceed one finger’s breadth anterior to the greater sciatic notch, and for the ischial cut, the chisel should advance at the level of one finger’s breadth below the distal joint edge. These landmarks can be more reliably determined when the quadrilateral plate is approached medially and directly visualized by the modified Stoppa approach.

Major arterial bleeding during PAO has been reported in previous literature. Thawrani et al. [29] reported a case with excessive arterial bleeding from the medial surface of the ilium after periosteal elevation that was not controllable by standard intraoperative techniques, and required an urgent embolization. Osteotomy of the superior pubic ramus has a potential risk of vascular injury due to its close relation with neurovascular structures [30]. Kambe et al. [31] measured the distance from the base of the superior pubic ramus to the obturator artery. The distance to the intrapelvic entry portal of the obturator canal was measured in men at 33.4 mm (range: 26–40 mm) and in women at 27.2 mm (range: 19–31 mm).

To avoid such complications, Wall et al. [32] and Inan et al. [30] recommended endoscopy-assisted PAO to visualize the inner aspect of the pelvis and minimize the risk of vascular injury. In the former study, the obturator nerve and artery lay 4–8 mm posterior to the superior pubic ramus making them at risk during an osteotomy. Utilizing the modified Stoppa intrapelvic approach, both structures can be protected by direct visualization and retraction.

The corona mortis has been previously identified as a source of severe and potentially fatal hemorrhage during orthopedic pelvic surgery. These vascular connections are not routinely visualized during classic Bernese PAO performed through the anterior approach, placing them at risk during the pubic osteotomy or placement of aberrant Hohman’s retractors. A magnetic resonance-based study by Hu et al. [33] identified corona mortis in 75% of patients undergoing PAO. They identified their most common location approximately 8.3 ± 3.8 mm medial and 11.1 ± 5.3 mm caudal from the anterosuperomedial edge of the iliopectineal eminence. Identification and ligation of corona mortis during the modified Stoppa approach help to minimize potential complications related to its injury.

Other complications that might be encountered when performing PAO with Smith-Peterson approach is damage to the femoral or sciatic nerves as a result of direct injury, excessive retraction, or aberrant Hohman placement [33]. These structured are rarely encountered during the modified Stoppa approach and the current study did not report such complications.

Utilizing modified Stoppa approach for PAO is infrequently discussed in literature. Klahs et al. [34] reported 2 cases where they utilized modified Stoppa approach for PAO and demonstrated the same benefits of enhanced visualization and reduced X-ray exposure. They recommended the utilization of the novel radiolucent retractors to provide adequate soft-tissue protection and not inhibiting the fluoroscopic with additional light source and suction port. Similarly, Saied et al. [35] conducted PAO through modified Stoppa approach on a series of 8 patients. Their data demonstrated significant improvement of the mean HHS from 66.8 to 92.7 points, and the mean LCEA from 13.12° to 28.37°.

In comparison to the classic Bernese anterior approach, performing PAO through modified Stoppa approach does not provide exposure to the hip joint capsule. Hence, intra-articular procedures if indicated to be performed may require a simultaneous (or staged) anterior approach to the joint or arthroscopy. These include osteochondroplasty for cam lesions, or labral repair. In addition, the modified Stoppa approach itself did not provide full exposure to perform the iliac cut, a lateral window was performed providing exposure for the lateral cut, and to allow for acetabular fragment manipulation and fixation.

In conclusion, Ganz PAO can be safely conducted through the modified Stoppa approach, providing direct exposure to the osteotomized surfaces, protects susceptible neuro-vascular structures. The procedure is associated with low operative time and blood loss and may reduce the overall morbidity of the procedure. Ganz PAO may be strongly considered as one of the expanding indications of modified Stoppa approach.

## Conflict of interest

All authors declare that they have no conflict of interest.

## Funding

No Funds have been received for this study.

## Ethical approval

“All procedures performed in studies involving human participants were in accordance with the ethical standards of the institutional and/or national research committee and with the 1964 Helsinki declaration and its later amendments or comparable ethical standards.” The study was approved by our institution’s Ethical Committee of Scientific Research.

## Informed consent

Informed consent was obtained from all individual participants included in the study. All parents consented and were informed that data concerning this study would be submitted for publication.

## Authors contributions

*Mostafa M. Baraka*: conceived and designed the study, drafted the manuscript, particularly involved in literature search, one of the main operating surgeons involved in analysis, and interpretation of data, reviewed the final manuscript critically for important intellectual content and approved the final version. *Mahmoud M. Abdelwahab*: patients follow-up, functional evaluation, acquisition, analysis, and interpretation of data, reviewed the final manuscript critically for important intellectual content and approved the final version. *Haitham E. Sallam*: drafted the manuscript, particularly involved in literature search, one of the main operating surgeons involved in analysis, and interpretation of data, reviewed the final manuscript critically for important intellectual content and approved the final version.
